# Traction esophageal diverticulum: a rare cause of gastro-intestinal bleeding

**DOI:** 10.1186/2193-1801-1-50

**Published:** 2012-11-21

**Authors:** Umashankar K Ballehaninna, Jason P Shaw, Igor Brichkov

**Affiliations:** 1 Department of Surgery, Maimonides Medical Center, Brooklyn, New York 11219 USA; 2 Department of Thoracic Surgery, Maimonides Medical Center, 4802 10th Avenue 4th Floor, Brooklyn, New York 11219 USA

## Abstract

Esophageal diverticula are uncommon lesions that are usually classified according to their location (cervical, thoracic, or epiphrenic), or underlying pathogenesis (pulsion or traction), and their morphology (true or false).The majority of esophageal diverticula are acquired lesions that occur predominantly in elderly adults. Pulsion, or false, diverticula are the most commonly encountered type of esophageal diverticula noticed at the level of cricopharyngeus muscle, occur as a localized outpouchings that lacks a muscular coat, and as such their wall is formed entirely by mucosa and submucosa.

True, or traction, esophageal diverticulum (TED) is seen in the middle one third of the thoracic esophagus in a peribronchial location, occurs secondary to mediastinal inflammatory lesions such as tuberculosis or histoplasmosis. The resultant desmoplastic reaction in the paraesophageal tissue causes full thickness pinching on the esophageal wall, producing a conical, broad-mouthed true diverticulum. They often project to the right side because subcarinal lymph nodes in this area are closely associated with the right anterior wall of the esophagus.

TED usually presents with symptoms such as dysphagia, postural regurgitation, belching, retrosternal pain, heartburn, and epigastric pain. As in patients with pharyngoesophageal (Zenker’s) diverticula, pulmonary symptoms are often present but underestimated in TED patients. These symptoms range from mild nocturnal cough to life-threatening massive aspiration. In this particular report we describe a rare case of TED presenting as a symptomatic upper gastrointestinal bleeding. Diagnostic evaluation of TED includes chest X-ray, barium esophagogram and manometry. A significant proportion of lower esophageal diverticula are associated with motility disorders. Management of TED include treating the underlying cause sometimes a surgical resection of diverticulum along with esophageal myotomy is necessitated in symptomatic patients.

## Traction esophageal diverticulum: a rare cause of gastro-intestinal bleeding

**Figure 1:**
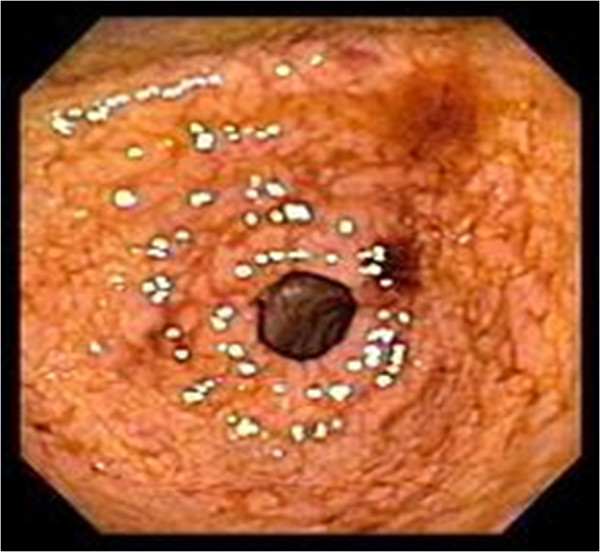
**An upper GI endoscopy revealed ulcerated esophageal diverticulum with minimal bleeding.**

**Figure 2:**
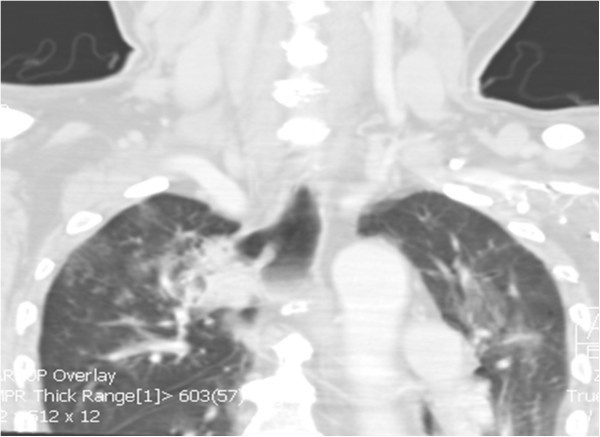
**CT scan of the chest was suggestive of an esophageal diverticulum communicating with apical segment of right upper lobe.**

**Figure 3:**
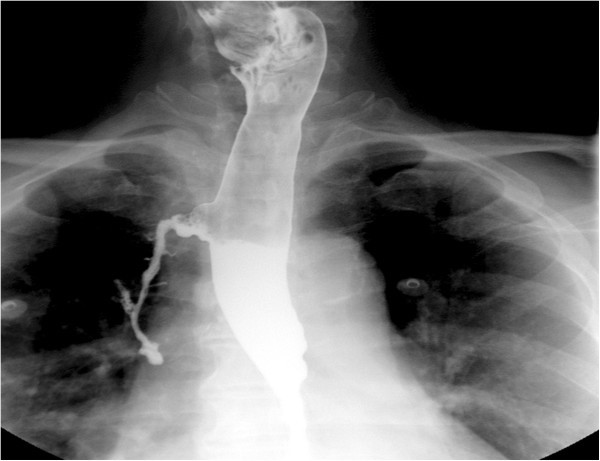
**Barium esophagram revealing traction esophageal diverticulum with communication into right upper lobe segmental bronchi.**

A 61-year-old male with multiple co-morbidities including atrial fibrillation managed with beta blockers and anticoagulation presented with recurrent hemetemesis and melena. After resuscitation, an upper endoscopy revealed a large esophageal diverticulum 22 cm from the incisors with ulceration and minimal bleeding (Figure 1). The patient also had recurrent cough and a chest x-ray showed a right upper lobe infiltrate. A subsequent CT scan of the chest suggested an esophageal traction diverticulum and erosion into the apical segment of right upper lobe. A barium esophagram confirmed a bronchoesophageal fistula (Figures 2 and 3). Simultaneous bronchoscopy and esophagoscopy revealed thick mucoid secretions and a fistula emanating from the esophageal diverticulum. Both endobronchial and esophageal biopsies revealed mucoid cells and chronic inflammatory changes. In view of this patient’s extensive co-morbidities, nasoesophageal and percutaneous endoscopic gastrostomy tubes were placed to aid healing of the fistula. A repeat contrast study performed after 3 months revealed complete resolution of the fistula and a persistent diverticulum.

Traction esophageal diverticula (TED) are true diverticula that occur as a result of contracture from chronic inflammation involving mediastinal structures in close proximity to the esophageal wall. Most TEDs occur in the mid esophagus. Common causes include tuberculosis, histoplasmosis and malignancy (Do Nascimento et al. 2006). TEDs usually present with dysphagia or recurrent aspiration. We describe here a rare presentation of upper gastro-intestinal bleeding resulting from ulceration and fistulization of the TED. Symptomatic TED are managed by local excision of the diverticulum via thoracotomy or thoracoscopy with concomitant treatment of the underlying inflammatory cause. Bronchoesophageal fistulae all require treatment by division of the fistula and reinforcement of the esophageal repair with a pedicled graft (López et al. 2003). Non-operative management with salivary diversion and enteral feeding may be successful in selected patients who are not candidates for surgical repair.
